# Posterior Corneal Elevation after Small Incision Lenticule Extraction for Moderate and High Myopia

**DOI:** 10.1371/journal.pone.0148370

**Published:** 2016-02-10

**Authors:** Yu Zhao, Meiyan Li, Jing Zhao, Michael C. Knorz, Ling Sun, Mi Tian, Xingtao Zhou

**Affiliations:** 1 Department of Ophthalmology, Eye and ENT Hospital, Myopia Key Laboratory of the Health Ministry, Fudan University, Shanghai, PR China; 2 Medical Faculty Mannheim of Heidelberg University, Mannheim, Germany; School of Ophthalmology and Optometry and the Affiliated Eye Hospital, Wenzhou Medical University, CHINA

## Abstract

**Purpose:**

To investigate the changes of posterior corneal elevation after small incision lenticule extraction (SMILE) for moderate and high myopia.

**Methods:**

In this prospective study, fifty consecutive eyes of thirty patients (10 male, 20 female) who underwent SMILE for myopia and myopic astigmatism were included. Eyes were divided in two groups based on the preoperative spherical equivalent refraction: high myopia group (32 eyes, range -6.25D to -10.00D) and moderate myopia group (18 eyes, range -3.00D to -6.00D). Posterior corneal surfaces were measured by a Scheimpflug camera (Pentacam, Oculus Germany) preoperatively and 1 month, 3 months, 6 months and 12 months postoperatively. Posterior central elevation (PCE) and posterior mean elevation (PME) at 17 predetermined points in the central-4mm area above the best-fit sphere were analyzed.

**Results:**

No significant difference in the amount of posterior corneal elevation changes in the high myopia group was noted over time (P = 0.23 and P = 0.94 for PCE and PME, respectively). Similarly, the changes in the moderate myopia group before and after SMILE were not significant either (P = 0.34 and P = 0.40 for PCE and PME). A statistically significant correlation was found between the residual bed thickness and the shift of the PCE in the high myopia group at 12 months postoperatively (r = 0.53, P = 0.01).

**Conclusions:**

The results of this study suggest that the posterior corneal surface remain stable within one year after SMILE for both moderate and high myopia. The changes of PCE correlate to the residual bed thickness for high myopia. Long-term changes of posterior corneal surface need further investigation.

## Introduction

Laser refractive surgery has been widely accepted for correction of refractive errors throughout the world. In the surgery, laser is used to reshape the anterior surface of the cornea by removing the corneal tissue in order to improve visual acuity. However, the surgery may weaken the cornea and might increase the risk for iatrogenic keratectasia [1,2]. As long as the anterior corneal surface is influenced by the procedure, analyzing changes in the posterior corneal surface is important for evaluation of corneal stability. Based on relevant researches, the alteration of the posterior corneal elevation in the early postoperative stage of refractive surgery is accurate in representing the posterior corneal shape and evaluating the corneal stability [3,4].

Femtosecond laser is gaining popularity in the field of refractive surgery in recent years. Femtosecond lenticule extraction (FLEx) requires no excimer laser or microkeratome, which creates both the lenticule and the flap. Small incision lenticule extraction (SMILE) is a variation of FLEx and has been defined as a flap-free corneal refractive surgery. SMILE creates an incision with a width of 2mm to 5mm, through which the refractive lenticule is separated and removed. As no flap is created, most anterior stromal lamellae remain intact, it is anticipated that SMILE can maintain a better biomechanical structure of corneal surface [5,6].

In this study, we analyzed posterior corneal elevation after SMILE in moderate and high myopia to investigate the potential change in corneal stability.

## Methods

### Subjects

In this prospective, non-randomized case series, 50 consecutive eyes of 30 patients who underwent SMILE from November 2011 to April 2012 at the Department of Ophthalmology, Eye and ENT Hospital of Fudan University (Shanghai, People’s Republic of China) were included. Eyes were divided into two groups based on the preoperative manifest spherical equivalent: high myopia group (32 eyes; range, -6.25D to -10.00D; mean -7.56±0.79D) and moderate myopia group (18 eyes; range, -3.00D to -6.00D; mean -4.99±0.65D). All participants in the study were healthy and met the inclusion criteria for refractive surgery. Each patient signed the informed consent after the details of the study were fully explained. The Ethical Committee of the Fudan University EENT Hospital Review Board approved the study protocol. All patients referred to the department for refractive surgery received a comprehensive ophthalmologic preoperative examination including slit-lamp examination, uncorrected distance visual acuity (UDVA), spherical equivalent refraction, intraocular pressure and Pentacam Imaging examinations. Detailed data are shown in Table 1.

**Table 1:** Patient Demographic Information (Mean ± Standard Deviation). SE: spherical equivalent; TCT: Thinnest Corneal Thickness; AD: Ablation Depth; RBT: Residual Bed Thickness.

	High myopia	Moderate myopia
Age (year)	29.34±7.10	28.17±5.76
Pre-op SE (D)	-7.56±0.79	-4.99±0.65
Pre-op TCT (μm)	551.34±33.07	542.44±24.77
AD (μm)	144.22±11.68	105.39±13.79
RBT (μm)	307.13±36.57	337.06±30.01

### Surgical Procedures

**Fig 1:**
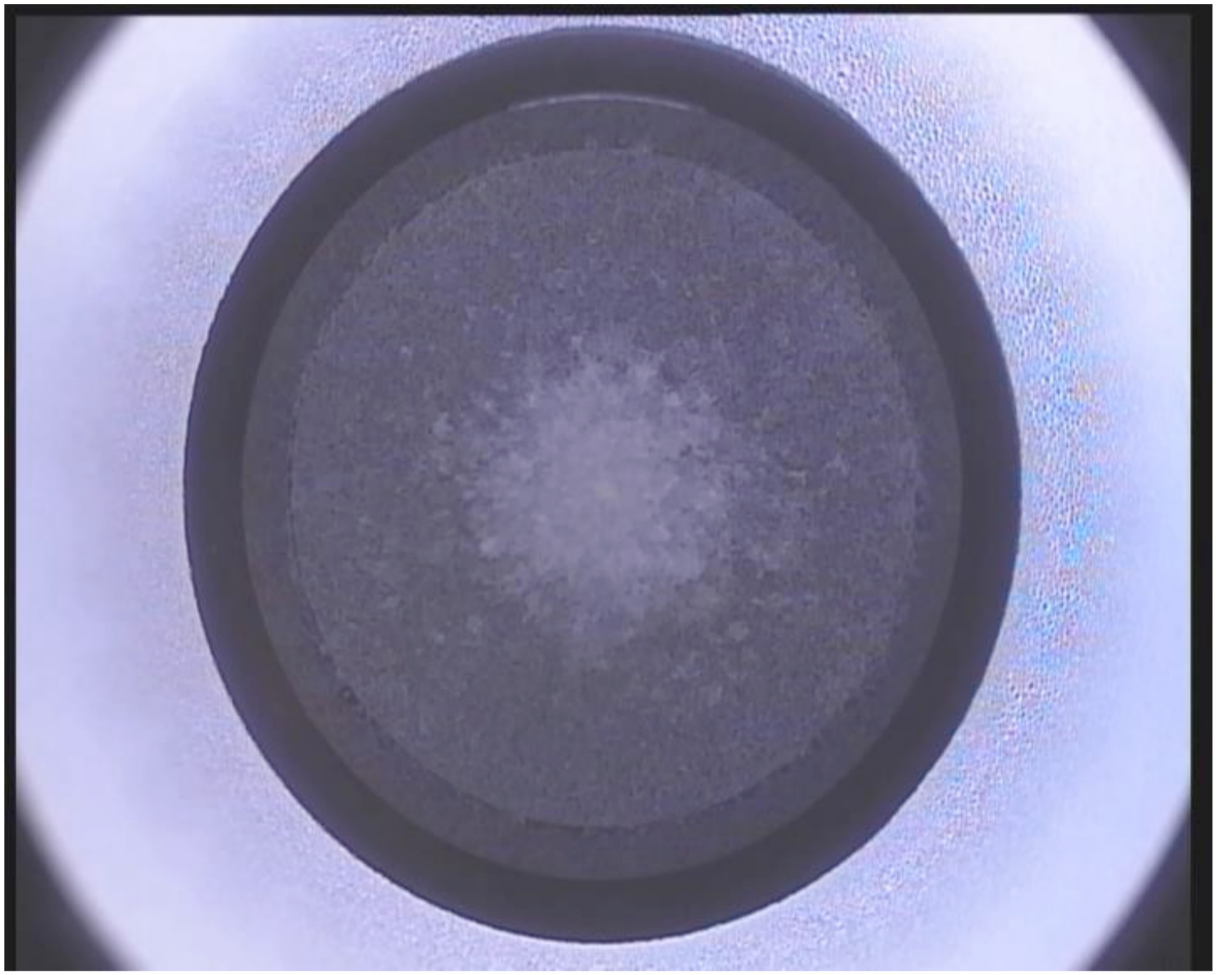
The VisuMax femtosecond laser system cuts the back of the refractive lenticule followed by its front surface, and finally completes the side incision.

**Fig 2:**
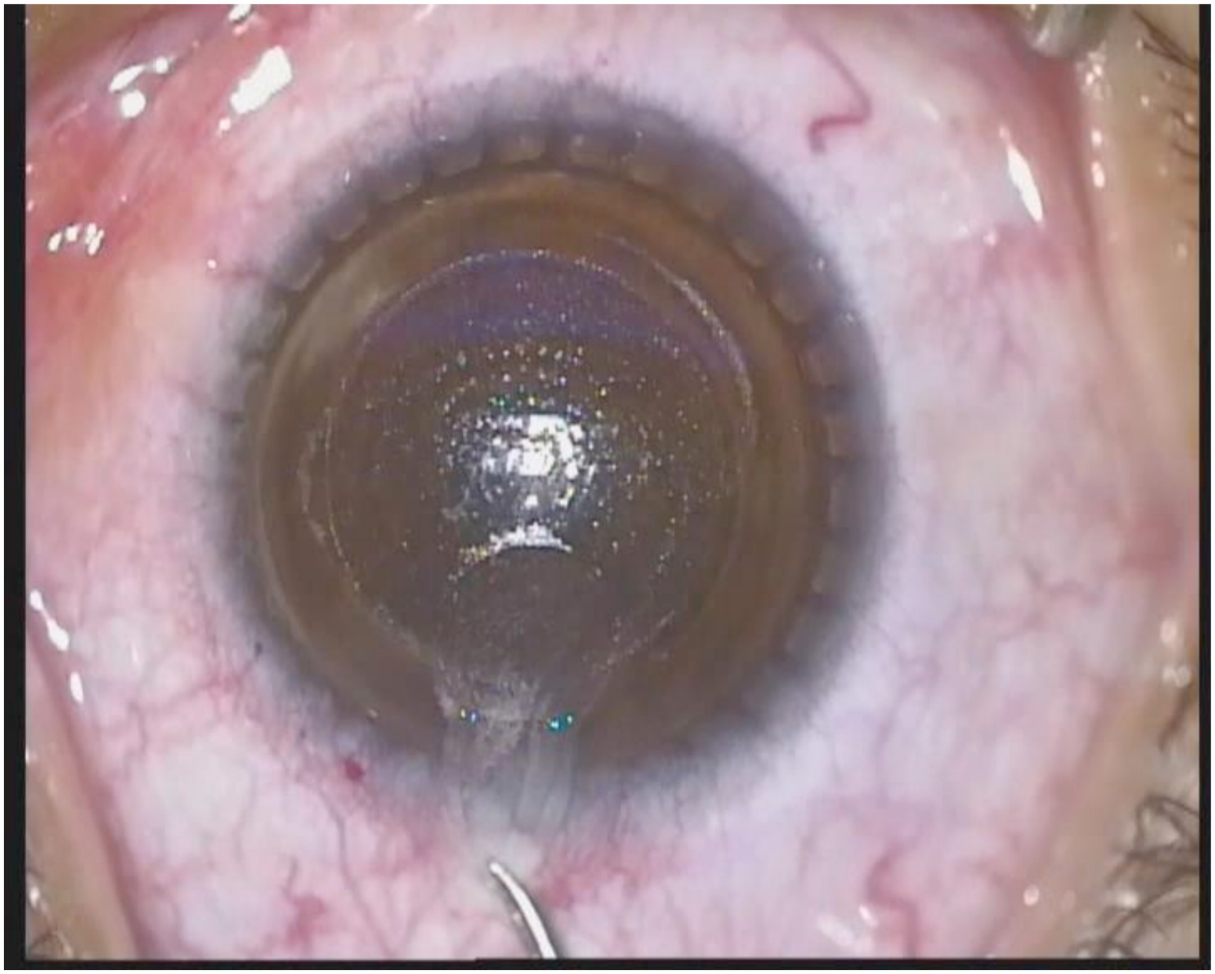
The lenticule being dissected and removed manually using forceps.

The VisuMax femtosecond laser system (Carl Zeiss Meditec AG, Germany) was used to perform SMILE in all patients. SMILE was performed with topical anesthesia using oxybuprocaine tetrachloride applied 2 to 3 minutes before the operation. The patient was positioned under the curved contact glass of the system and required to fixate on the target. The intended thickness of the cap was settled at 100μm. The surgical procedure was described by Yao P previously [7]. After the posterior and anterior surface of the refractive lenticule were finished scanning, the side cut was created at 12-o’clock with a width of 2mm (Fig 1). The refractive lenticule was subsequently separated through the side-cut incision and manually removed (Fig 2).

### Pentacam Scheimpflug

Patients underwent Pentacam Scheimpflug imaging (Oculus GmbH, Wetzlar, Germany) preoperatively and at 1, 3, 6, 12 months after SMILE. All Scheimpflug scans were performed by the same trained operator. The patient was asked to position properly and fixed on the blue beam after blinking twice. After realization of the alignment, the instrument automatically captured 25 rotational Scheimpflug images within 2 seconds, which was the point the light crossed the cornea from the fovea to the device. In order to avoid miscalculations of poor imaging quality, the quality of the measurement results were under specification window inspection; only results with “OK” statements were accepted. The examination was duplicated if the comment was marked yellow or red. Only maps with at least 10 mm of corneal coverage and no deduced data in the central 9-mm zone were accepted.

### Data Collection

Elevation data of the posterior corneal surfaces were obtained using the Pentacam software. To calculate elevation data across all pre- and postoperative examinations, the reference best-fit sphere (BFS) was defined in the central 8.0-mm region of the preoperative cornea. Calculated values were posterior central elevation (PCE) and posterior mean elevation (PME) above BFS. Corneal PME data were obtained from 17 points in the central 4-mm zone as follows: 1 point at the center, 8 points at 1 mm distance from the center along 0°, 45°, 90°, 135°, 180°, 225°, 270° and 315° meridians (0° defined as the horizontal semi-meridian on the right, and rotating counterclockwise in both eyes), and the other 8 points at 2 mm distance from the center along the identical semi-meridians. PME was calculated as the mean value in corneal posterior elevation for these 17 points. The ΔPCE and ΔPME were defined by subtracting preoperative data from postoperative data (difference elevation map). By this way, we could confirm that is was always the same point observed in different times. As such, an ectatic change of the posterior corneal surface would be a positive number. The change in elevation was considered to be the shift of the posterior corneal surface.

Data of the preoperative thinnest corneal thickness were obtained from the Pentacam. Values of ablation depth were exported from the VisuMax program. Subtracting cap thickness (100μm) and ablation depth from preoperative thinnest corneal thickness, we got value of estimated residual bed thickness (RBT).

### Statistical Analysis

Descriptive results contained the mean and the standard deviation. Kolmogorov-Smirnow normality test was executed for all data above all. Bartletts test for equal variances was applied subsequently. The one-way analysis of variance (ANOVA) for repeated measures was employed when comparing changes of elevation values within the group. Pearson’s linear correlation was used to investigate the linear relationships between the change of the posterior corneal elevation and preoperative thinnest corneal thickness, ablation depth, and RBT. Statistical analyses were performed using SPSS ver.19.0 (SPSS Inc, Chicago, IL, USA). A P value< 0.05 was considered to indicate a statistically significant difference.

## Results

All the surgeries were completed without complications. No case of keratectasia was observed in our series. An UDVA of 20/25 or better was achieved in 100% of eyes at 1-day follow-up. Twelve months postoperatively, 100% (50 eyes) of eyes had an UDVA of 20/20 or better, 96% (48 eyes) of eyes were within ±0.5D and 100% (50 eyes) were within ±1.0D. There was no regression of more than -0.5D toward the final 12-month visit in all eyes.

**Fig 3:**
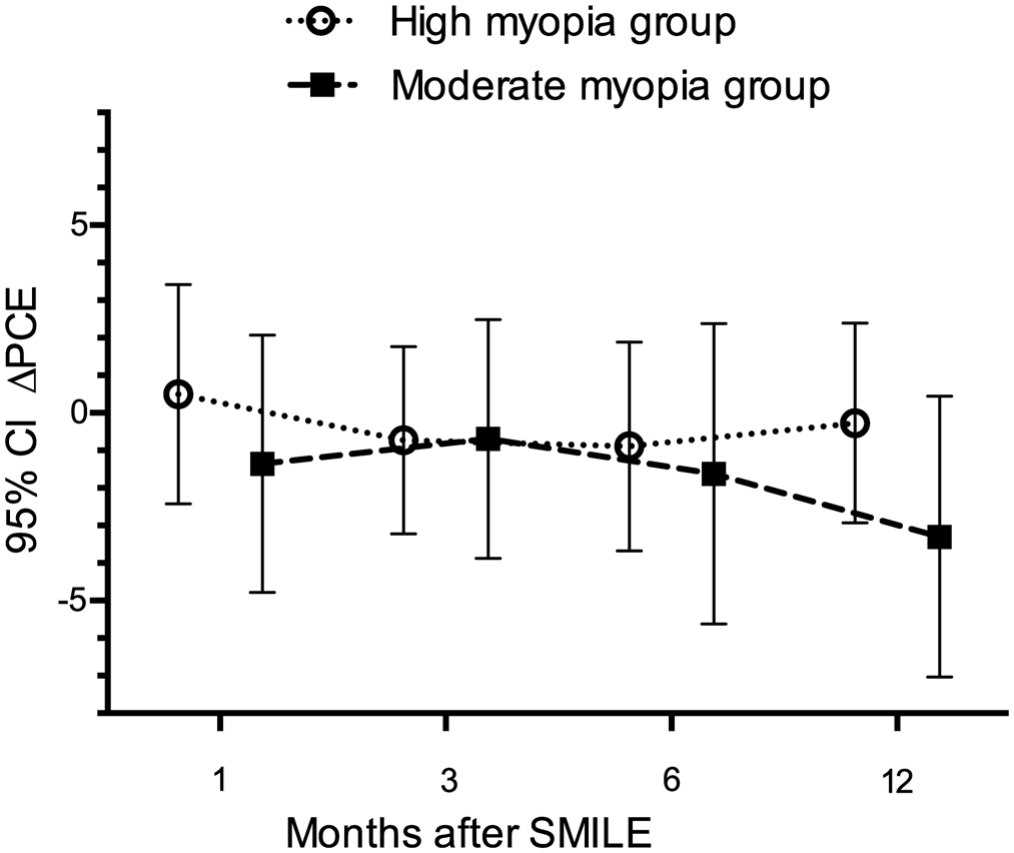
Changes in posterior central elevation (ΔPCE) at 1 month, 3 months, 6 months, 12 months after SMILE in various groups. Circle: ΔPCE at different follow-ups in high myopia group; square: ΔPCE at different follow-ups in moderate myopia group. No significant difference was noted in ΔPCE in both groups.

**Fig 4:**
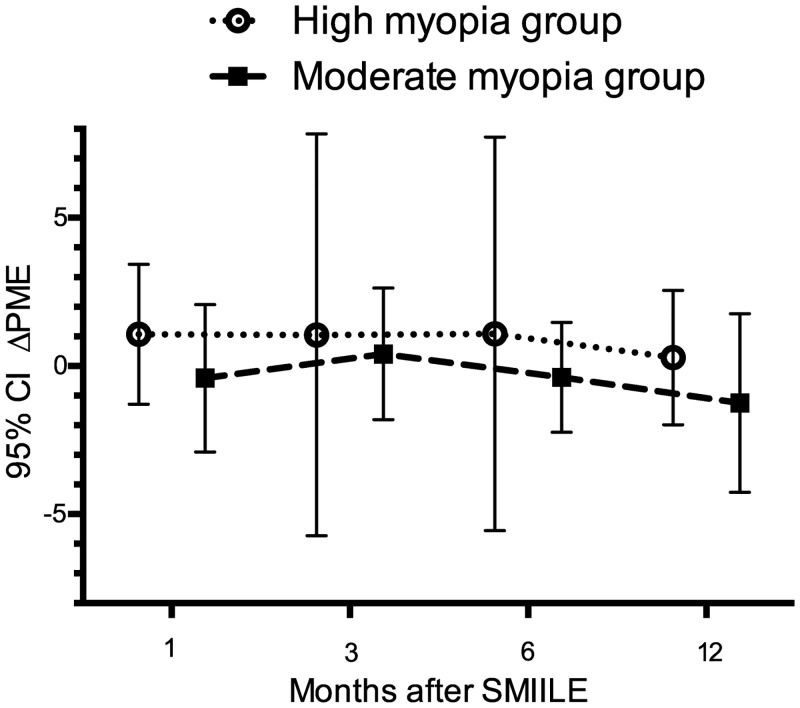
Changes in posterior mean elevation (ΔPME) at 1 month, 3 months, 6 months, 12 months after SMILE in various groups. Circle: ΔPME at different follow-ups in high myopia group; square: ΔPME at different follow-ups in moderate myopia group. No significant difference was noted in ΔPME in both groups.

The average PCE changes (ΔPCE) at 1, 3, 6, and 12 months after SMILE in the high myopia group were 0.50±2.92μm, -0.73±2.49μm, -0.89±2.78μm, -0.27±2.66μm, respectively. None of these differences was statistically significant (*F* = 1.47, *P* = 0.23; Fig 3). Mean PME change (ΔPME) in the high myopia group at 1 month after SMILE was 1.07±2.36μm, at 3 months was 1.05±6.78μm, at 6 months was 1.09±6.64μm, and at 12 months was 0.29±2.77μm. The changes in ΔPME before and after 1, 3, 6, 12 months in the high myopia group was not significant either (*F* = 0.13, *P* = 0.94; Fig 4).

As shown in Fig 3, mean values of ΔPCE in the moderate myopia group were -1.35±3.43μm at 1 month, -0.69±3.18μm at 3 months, -1.62±4.0μm at 6 months and -3.30±3.74μm at 12 months. Average ΔPME differences at 1, 3, 6, and 12 months after SMILE were -0.41±2.49μm, 0.41±2.22μm, -0.38±1.86μm, and -1.25±3.01μm, respectively. There was no statistically significant difference in ΔPCE or in ΔPME in the moderate myopia group (*F* = 1.14, *P* = 0.34 for ΔPCE, Fig 3; *F* = 1.01, *P* = 0.40 for ΔPME, Fig 4). The power for the statistical tests was between 0.25 and 0.40 for ΔPCE and ΔPME in both groups.

**Fig 5:**
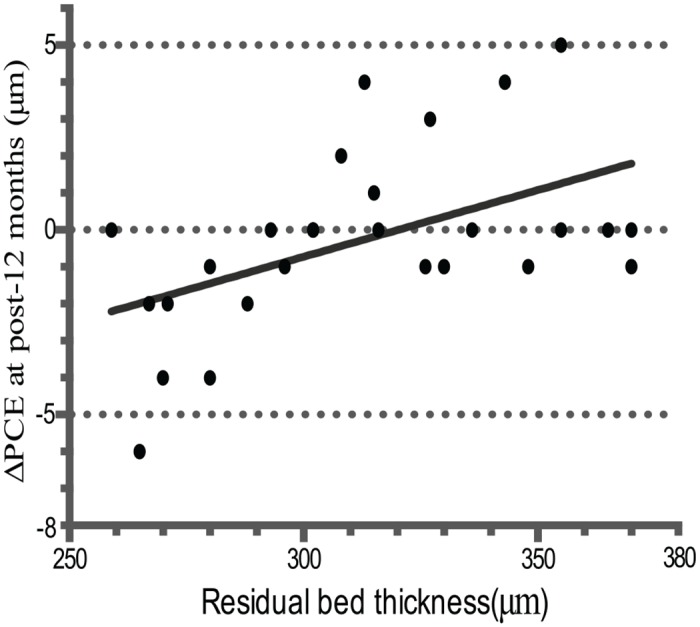
Significant correlation was found between the change of posterior central elevation (ΔPCE) at 12 months after SMILE and residual bed thickness (RBT) in the high myopia group (*r* = 0.53, *P* = 0.01).

In the high myopia group, RBT correlated significantly with ΔPCE at 12 months postoperatively (*r* = 0.53, *P* = 0.01, Fig 5). Additionally, preoperative thinnest corneal thickness and ablation depth correlated significantly with ΔPCE at 12 months after surgery (*r* = 0.43, *P* = 0.045; *r* = -0.44, *P* = 0.04). However, no correlation was noted between the ΔPCE and RBT, preoperative thinnest corneal thickness, and ablation depth at other follow-up visits. Besides, there was no linear relationship between changes in ΔPME and preoperative thinnest corneal thickness, ablation depth, or RBT. What’s more, there was no statistical significant linear relationship between changes in posterior corneal elevation and preoperative thinnest corneal thickness, ablation depth, or RBT in the moderate myopia group.

## Discussion

Laser refractive surgery is an effective method for the correction of myopia by removing some corneal tissue. The postoperative corneal stability is a focal point of research because the corneal may become weaker postoperatively. The change of the posterior corneal surface is considered to be an early indicator of ectatic change of the cornea after refractive surgery. Elevation data is more accurate in representation of the posterior corneal surface than curvature data because it is independent of orientation, axis and not affected by tear film. There have been many reported studies on evaluating changes of corneal posterior elevation in the certain area after refractive surgeries [8–14].

SMILE is a new surgical technique of “flapless” refractive surgery. During the surgery, only femtosecond laser is used to perform the whole procedure, thus reducing surgical time and cost. Recent publications suggest that SMILE has excellent predictability, safety, and efficacy [15–17]. In addition, SMILE is likely to weaken corneal biomechanics less than laser in situ keratomileusis (LASIK) and make posterior corneal surfaces more stable in postoperative eyes. To the best of our knowledge, the current study is the first to report the posterior corneal surface changes after SMILE.

In this study, analysis of the elevation data was restricted to the central 4-mm area to ensure that the standard deviation of elevation was kept at a low level. Two parameters were used to evaluate possible changes of the posterior corneal surface: posterior central elevation (PCE) and posterior mean elevation (PME).

The mean changes of PCE and PME in both the high myopia group and the moderate myopia group were no significant difference. In addition, all changing values at different follow-up points were within the measurement noise of the device as the reported Pentacam range of error was ±5μm [12]. The results of this study indicated that SMILE may cause no significant change in the central posterior cornea surface.

Previous studies have showed that no posterior corneal protrusion was observed using Pentacam after LASIK, epipolis laser in situ keratomileusis (Epi-LASIK) or photorefractive keratectomy (PRK). Although the corneal posterior surfaces after SMILE seemed as stable as those after excimer laser surgeries, it is reasonable to believe that SMILE benefits the corneal biomechanics more than other refractive surgeries. Biomechanical parameters such as corneal hysteresis (CH) and corneal resistance factor (CRF) were showed to be significantly higher in post-SMILE eyes than in post-LASIK eyes [5]. Dong Z also reported that total stromal tensile strength was considerably higher after SMILE than both PRK and LASIK [18]. The advantage of SMILE could be due to the following reasons: First, photo disruption by a femtosecond laser as compared to ablation by an excimer laser may cause less tissue damage in SMILE [19,20]; Second, SMILE could cause less epithelial trauma due to the small incision, and more anterior lamellae remain intact after SMILE [18,19].

A statistical correlation was found between RBT, preoperative thinnest corneal thickness, ablation depth and the shift of PCE in the high myopia group at 12 months after SMILE. Previous studies have demonstrated that eye with thinner cornea or deeper ablation was more likely leading to a shift of the posterior corneal surface [21]. Wang et al found that posterior corneal protrusion was correlated to RBT, and the risk of keratectasia may be increased if RBT was thinner than 250μm [22]. In our study, none of the eyes had a residual corneal thickness of less than 250μm. There was no case of keratectasia. Published studies suggested that SMILE could allow deeper corrections in the corneal stroma since it presents less biomechanical risk to the residual bed of corneas comparing with LASIK [23]. As eyes after SMILE have more stable posterior corneal surfaces, the security residual bed thickness may be lower than 250μm in post-SMILE eyes [23]. Further investigations on this issue should be conducted to confirm it.

Interestingly, patients undergoing PRK or LASIK were found to experience forward displacements of the posterior corneal surfaces in the early studies [21,24]. However, most of these reports were based on Orbscan posterior maps. Many reports found that Orbscan may overestimate pachmetry values in normal corneas compared with ultrasound pachymetry [25,26]. Using the more recently developed Pentacam, the protrusion of the posterior cornea after LASIK was reported to be significantly lower of 17 to 55μm than the values measured by Orbscan [27–29]. Pérez-Escudero conducted a validation experiment for the Pentacam and found the data can be obtained with high repeatability [30]. These findings suggest that the Pentacam is precise in measuring posterior corneal elevation.

With respect to the findings of this study, studies with a larger patient base are required to validate the stability of the posterior corneal surface after SMILE. As this research limits the measurement of posterior elevation in the central 4.0-mm zone, calculation of the posterior elevation in a wider area may provide more insights.

## References

[1] Seiler T, Quurke AW (1998) Iatrogenic keratectasia after LASIK in a case of forme fruste keratoconus. J Cataract Refract Surg 24: 1007–1009. 9682124 10.1016/s0886-3350(98)80057-6

[2] Pallikaris IG, Kymionis GD, Astyrakakis NI (2001) Corneal ectasia induced by laser in situ keratomileusis. J Cataract Refract Surg 27: 1796–1802. 11709254 10.1016/s0886-3350(01)01090-2

[3] Ciolino JB, Belin MW (2006) Changes in the posterior cornea after laser in situ keratomileusis and photorefractive keratectomy. J Cataract Refract Surg 32: 1426–1431. 16931251 10.1016/j.jcrs.2006.03.037

[4] Ciolino JB, Khachikian SS, Cortese MJ, Belin MW (2007) Long-term stability of the posterior cornea after laser in situ keratomileusis. J Cataract Refract Surg 33: 1366–1370. 17662425 10.1016/j.jcrs.2007.04.016

[5] Wu D, Wang Y, Zhang L, Wei S, Tang X (2014) Corneal biomechanical effects: small-incision lenticule extraction versus femtosecond laser-assisted laser in situ keratomileusis. J Cataract Refract Surg 40: 954–962. 10.1016/j.jcrs.2013.07.056 24751146

[6] Blum M, Kunert K, Schroder M, Sekundo W (2010) Femtosecond lenticule extraction for the correction of myopia: preliminary 6-month results. Graefes Arch Clin Exp Ophthalmol 248: 1019–1027. 10.1007/s00417-009-1293-1 20130899

[7] Yao P, Zhao J, Li M, Shen Y, Dong Z, Zhou X. (2013) Microdistortions in Bowman's layer following femtosecond laser small incision lenticule extraction observed by Fourier-Domain OCT. J Refract Surg 29: 668–674. 23938095 10.3928/1081597X-20130806-01

[8] Belin MW, Zloty P (1993) Accuracy of the PAR corneal topography system with spatial misalignment. CLAO J 19: 64–68. 8453756 10.1097/00140068-199301000-00012

[9] Nilforoushan MR, Speaker M, Marmor M, Abramson J, Tullo W, Morschauser D, et al (2008) Comparative evaluation of refractive surgery candidates with Placido topography, Orbscan II, Pentacam, and wavefront analysis. J Cataract Refract Surg 34: 623–631. 10.1016/j.jcrs.2007.11.054 18361985

[10] Mihaltz K, Kovacs I, Takacs A, Nagy ZZ (2009) Evaluation of keratometric, pachymetric, and elevation parameters of keratoconic corneas with pentacam. Cornea 28: 976–980. 10.1097/ICO.0b013e31819e34de 19724217

[11] Khairat YM, Mohamed YH, Moftah IA, Fouad NN (2013) Evaluation of corneal changes after myopic LASIK using the Pentacam(R). Clin Ophthalmol 7: 1771–1776. 10.2147/OPTH.S48077 24043925 PMC3772758

[12] Grewal DS, Brar GS, Grewal SP (2011) Posterior corneal elevation after LASIK with three flap techniques as measured by Pentacam. J Refract Surg 27: 261–268. 10.3928/1081597X-20100618-01 20672773

[13] Zhang L, Wang Y (2010) The shape of posterior corneal surface in normal, post-LASIK, and post-epi-LASIK eyes. Invest Ophthalmol Vis Sci 51: 3468–3475. 10.1167/iovs.09-4811 20207960

[14] Ha BJ, Kim SW, Kim SW, Kim EK, Kim TI (2009) Pentacam and Orbscan II measurements of posterior corneal elevation before and after photorefractive keratectomy. J Refract Surg 25: 290–295. 19370825 10.3928/1081597X-20090301-09

[15] Sekundo W, Kunert KS, Blum M (2011) Small incision corneal refractive surgery using the small incision lenticule extraction (SMILE) procedure for the correction of myopia and myopic astigmatism: results of a 6 month prospective study. Br J Ophthalmol 95: 335–339. 10.1136/bjo.2009.174284 20601657

[16] Sekundo W, Gertnere J, Bertelmann T, Solomatin I (2014) One-year refractive results, contrast sensitivity, high-order aberrations and complications after myopic small-incision lenticule extraction (ReLEx SMILE). Graefes Arch Clin Exp Ophthalmol 252: 837–843. 10.1007/s00417-014-2608-4 24647595

[17] Kunert KS, Melle J, Sekundo W, Dawczynski J, Blum M (2015) [One-year results of small incision lenticule extraction (SMILE) in myopia]. Klin Monbl Augenheilkd 232: 67–71. 25272083 10.1055/s-0034-1383053

[18] Reinstein DZ, Archer TJ, Randleman JB (2013) Mathematical model to compare the relative tensile strength of the cornea after PRK, LASIK, and small incision lenticule extraction. J Refract Surg 29: 454–460. 10.3928/1081597X-20130617-03 23820227

[19] Dong Z, Zhou X, Wu J, Zhang Z, Li T, Zhou Z, et al (2014) Small incision lenticule extraction (SMILE) and femtosecond laser LASIK: comparison of corneal wound healing and inflammation. Br J Ophthalmol 98: 263–269. 10.1136/bjophthalmol-2013-303415 24227802 PMC3913294

[20] Riau AK, Angunawela RI, Chaurasia SS, Lee WS, Tan DT, Mehta JS. (2011) Early corneal wound healing and inflammatory responses after refractive lenticule extraction (ReLEx). Invest Ophthalmol Vis Sci 52: 6213–6221. 10.1167/iovs.11-7439 21666235

[21] Baek T, Lee K, Kagaya F, Tomidokoro A, Amano S, Oshika T. (2001) Factors affecting the forward shift of posterior corneal surface after laser in situ keratomileusis. Ophthalmology 108: 317–320. 11158806 10.1016/s0161-6420(00)00502-9

[22] Wang Z, Chen J, Yang B (1999) Posterior corneal surface topographic changes after laser in situ keratomileusis are related to residual corneal bed thickness. Ophthalmology 106: 406–409; discussion 409–410. 9951499 10.1016/S0161-6420(99)90083-0

[23] Sinha Roy A, Dupps WJ Jr., Roberts CJ (2014) Comparison of biomechanical effects of small-incision lenticule extraction and laser in situ keratomileusis: finite-element analysis. J Cataract Refract Surg 40: 971–980. 10.1016/j.jcrs.2013.08.065 24857440 PMC6030688

[24] Kim H, Kim HJ, Joo CK (2006) Comparison of forward shift of posterior corneal surface after operation between LASIK and LASEK. Ophthalmologica 220: 37–42. 16374047 10.1159/000089273

[25] Chakrabarti HS, Craig JP, Brahma A, Malik TY, McGhee CN (2001) Comparison of corneal thickness measurements using ultrasound and Orbscan slit-scanning topography in normal and post-LASIK eyes. J Cataract Refract Surg 27: 1823–1828. 11709257 10.1016/s0886-3350(01)01089-6

[26] Modis L Jr., Langenbucher A, Seitz B (2001) Scanning-slit and specular microscopic pachymetry in comparison with ultrasonic determination of corneal thickness. Cornea 20: 711–714. 11588422 10.1097/00003226-200110000-00008

[27] Twa MD, Roberts C, Mahmoud AM, Chang JS Jr. (2005) Response of the posterior corneal surface to laser in situ keratomileusis for myopia. J Cataract Refract Surg 31: 61–71. 15721697 10.1016/j.jcrs.2004.09.032

[28] Hashemi H, Mehravaran S (2007) Corneal changes after laser refractive surgery for myopia: comparison of Orbscan II and Pentacam findings. J Cataract Refract Surg 33: 841–847. 17466859 10.1016/j.jcrs.2007.01.019

[29] Nishimura R, Negishi K, Saiki M, Arai H, Shimizu S, Toda I, et al (2007) No forward shifting of posterior corneal surface in eyes undergoing LASIK. Ophthalmology 114: 1104–1110. 17239440 10.1016/j.ophtha.2006.09.014

[30] Chen D, Lam AK (2007) Intrasession and intersession repeatability of the Pentacam system on posterior corneal assessment in the normal human eye. J Cataract Refract Surg 33: 448–454. 17321396 10.1016/j.jcrs.2006.11.008

